# Supplementary material for: A Critical View of the Application of the APEX Software (Aqueous Photochemistry of Environmentally-Occurring Xenobiotics) to Predict Photoreaction Kinetics in Surface Freshwaters
Source: Molecules. 2019 Dec 18;25(1):9. doi: 10.3390/molecules25010009 (PMC7017383; doi:10.3390/molecules25010009)
Supplement: Supplementary file 1 [file molecules-25-00009-s001.zip › 3.2.4_gcc-4.4.0.zip!/3.2.4_gcc-4.4.0/3.2.4_gcc-4.4.0/doc/octave/html/interpreter/Missing-Data.html]

Missing Data - Untitled


Next: String Objects,
Previous: Numeric Objects,
Up: Built-in Data Types


---

#### 3.1.2 Missing Data

It is possible to represent missing data explicitly in Octave using
`NA` (short for “Not Available”). Missing data can only be
represented when data is represented as floating point numbers. In this
case missing data is represented as a special case of the representation
of `NaN`.

— Built-in Function: **NA**  
— Built-in Function: **NA** (n)  
— Built-in Function: **NA** (n, m)  
— Built-in Function: **NA** (n, m, k, ...)  
— Built-in Function: **NA** (..., class)  
> Return a scalar, matrix, or N-dimensional array whose elements are all equal
> to the special constant used to designate missing values.
>
> Note that NA always compares not equal to NA (NA != NA).
> To find NA values, use the `isna` function.
>
> When called with no arguments, return a scalar with the value NA.
> When called with a single argument, return a square matrix with the dimension
> specified. When called with more than one scalar argument the first two
> arguments are taken as the number of rows and columns and any further
> arguments specify additional matrix dimensions.
> The optional argument class specifies the return type and may be
> either "double" or "single".
>
>
>
> **See also:** isna.

— Mapping Function: **isna** (x)  
> Return 1 for elements of x that are NA (missing) values and zero
> otherwise. For example,
>
> ```
>           isna ([13, Inf, NA, NaN])
>                => [ 0, 0, 1, 0 ]
> ```
>
>
>
>
> **See also:** isnan.
